# Why the Rise of Predatory Journals: View of the Prey

**DOI:** 10.1111/ans.70173

**Published:** 2025-05-07

**Authors:** David A. Clark, Michael Solomon

**Affiliations:** ^1^ Department of Surgery Royal Brisbane and Women's Hospital Herston Queensland Australia; ^2^ Department of Colorectal Surgery Royal Prince Alfred Camperdown New South Wales Australia

**Keywords:** author, predatory journals, publishing

## Abstract

For many authors, the publishing world is opaque and complex. With the rise of predatory journals, the sharks are circling inexperienced authors. There have been steps taken by publishers to mitigate the risk to scientific publishing, and this article seeks to approach the issue from the author's view.

## 1 Perspective Piece

In an interconnected world, consumers explore the internet for information on health conditions. Journals are the repository of robust, reliable, and peer reviewed publications. The rise of predatory journals undermines this long‐established covenant. Cursory peer‐review practices of predatory journals and open access create a veneer of respectability, and many readers will be inadequately equipped to determine scientific merit. The publication of poor‐quality manuscripts undermines the legitimate work produced by genuine authors and is a concern for truth in a world where truth and opinion are blurred.

The publication of the 2024 Asia Pacific Association of Medical Journal Editors (APAME) declaration on predatory journals [1] and the recent editorial in the British Medical Journal [2] show the topic is of great concern to the academic publishing industry. It is clear that the issue can only be approached by the involvement of all stakeholders: the publishers, the authors, and the institutions that use publication metrics as a measure of accomplishment.

### 1.1 How Authors May Feel

There can be considerable disappointment in the publication process from the author's perspective. The research project often requires a considerable timeline, and much effort is expended in obtaining research ethics and governance approval. By the time the study is completed, analyzed, and written up, the authors will be attached to the finished manuscript and might, unrealistically, expect that journals will welcome their work.

For many authors, the publishing world is opaque and complex. It is unclear how the reviewers are chosen and how they are vetted. The request from some mainstream journals to provide your own suggested reviewers is confusing, as that would present a clear conflict of interest. Timelines are uncertain and may not reflect what is reported by the journal. Into this space, predatory journals step and deliver high acceptance rates with perfunctory critical review. Whilst this comes with a financial price that authors may be willing to pay for career advancement, the price to the body of scientific publication is paid by the whole community.

### 1.2 Burden on Authors

“Respected Sir, we have genuinely tried many times…” Authors will recognize such emails as they overwhelm inboxes. Are email addresses gleaned from publicly available publications? The display of authors' email addresses in publications is problematic and authors may be reticent to provide their primary email address. Could the distribution of the infrequent but legitimate questions regarding publications be handled by journals themselves? This would reduce the reach of predatory journals to legitimate authors.

Authors are also peer reviewers, and their time is a finite resource. The model of content providers and content reviewers providing their time and effort pro bono has evolved out of the historical models of print publishing and the kudos of doing so. Predatory journals seek to leverage this established arrangement and consequently dilute the procurable reviewer resource. This will contribute to delays in manuscript review in legitimate journals.

### 1.3 Potential Solutions

Lists are available of predatory journals, but these lists are likely incomplete and, by definition, evolve rapidly to become obsolete. Rather than list the predatory journals, why not reverse the process and list only the reputable ones? There are bodies that do that: the World Association of Medical Editors (WAME), the International Committee of Medical Journal Editors (ICMJE), the Committee on Publication Ethics (COPE) and the website ThinkCheckSubmit.org. The journal ranking website, Scimago, provides ranking data but is supported by advertising. To make this premise function effectively, a charter of common ideals and standards would need to be agreed and set by all stakeholders, and bodies that use publication metrics (universities, health employers, colleges or training program selection committees) would have to adhere to these criteria and disregard or relegate to a lower tier publications in non‐listed journals. This would help the assessors in scoring candidates who have applied to training programs, employers for determining promotions, and importantly help prospective authors to discriminate robust journals as submission targets. This would de‐incentivize the predatory journal model.

Journal editors and authors share the commonly aligned goals of publication of high quality research and expert commentary to inform clinical and research practice. In a rapidly changing environment and with a thirst of the general population for reliable and trustworthy information, alongside the scientific community, the challenge is to maintain the high standards and democracy of knowledge without the erosion of trust that comes with poor quality publication practices. Alas, the prey becomes the pursuit of truth itself.

## Conflicts of Interest

The authors declare no conflicts of interest.
